# Benefit finding in chronic kidney disease patients receiving hemodialysis: a cross-sectional study

**DOI:** 10.1186/s12882-024-03480-7

**Published:** 2024-02-01

**Authors:** Jie Yang, Hong-Li Yan, Yong-Qi Li, Lei Zhang, Xiao-Yan Qiu, Yi-He Tian, Yan-Lin Gong, Chen-Ling Luo, Jing Wu, Jing Chu

**Affiliations:** 1 School of Nursing, Naval Medical University, Shanghai, China; 2 School of Health Services Management, Southern Medical University, Guangzhou, China; 3 Department of Pain Rehabilitation, Qingdao Special Servicemen Recuperation Center of PLA NAVY, Qingdao, China; 4 Trauma Surgery, Hospital of Chinese People’s Armed Police Force, Jiaxing, China; 5 Department of Nursing, Southern Medical University, Guangzhou, China

**Keywords:** Hemodialysis, Benefit finding, Social support, Coping style, Self-efficacy

## Abstract

**Background and objectives:**

The psychological problems of hemodialysis (HD) patients are prominent, and benefit finding (BF) have been proven beneficial to physical and mental health, fewer researchers explored BF in HD patients. The aim of this study was to investigate the current status of BF in patients with chronic kidney disease and to analyze the factors influencing it in order to provide a reference for subsequent interventions.

**Methods:**

A cross-sectional study was done on 246 HD patients by convenience sampling in the hemodialysis center of a 3 A hospital in Shanghai from March to September 2019. The measures include General Information Questionnaire, Benefit Finding Scale, Perceived Social Support Scale, General Self-efficacy Scale, and Simplified Coping Style scale.

**Results:**

The median (interquartile range, IQR) score of BF was 66 (IQR = 19) and it was lower compared with other chronic diseases. Significant differences in BF scores were found between different age groups, HD duration categories, and understanding degrees of HD. Taking BF as the dependent variable, the results of multiple linear regression analysis showed that age, duration of HD, family support, other support, positive coping, and self-efficacy entered the regression equation to explain 43.8% of the total variation. Social support played an indirect effect in the relationship between positive coping and BF, accounting for 54.1% of the total effect.

**Conclusion:**

The BF of HD patients is worrisome and affected by many factors. Medical staff could pay attention to the positive psychology of HD patients, and construct individualized interventions according to the influencing factors to improve their BF level and achieve physical and mental health.

**Supplementary Information:**

The online version contains supplementary material available at 10.1186/s12882-024-03480-7.

## Introduction

*Hemodialysis (HD)* is the most common treatment regime for patients with acute and chronic renal failure [1], and the proportion of HD patients worldwide increased by 43.1% in 2017 compared to 1990 [2]. China is one of the countries with the largest number of HD patients in the world. The corresponding number of Chinese prevalent dialysis patients in 2016 was approximately 578,000 [3], and the number continues to rise year by year. HD can prolong the survival time and improve the survival rate of patients with end-stage renal disease. However, HD patients experience a high burden of physical and emotional symptoms resulting in poor quality of life [4]. A study showed that long-term HD would lead to poorer mental health and declining quality of life in patients [5]. Some studies have shown that 41% of HD patients had symptoms of depression [6], and about 12.6% suffered from depression and anxiety at the same time [7]. These reports suggest an urgent need to attach importance to the psychological status of HD patients.

*Benefit Finding (BF)* was first put forward by Taylor in 1983 as an important and novel concept in positive psychology [8]. It refers to the perception of an individual’s positive response to adverse life events, manifested as positive cognitive and behavioral reactions [8]. BF had been explored and proven mainly in cancer patients [9–11], family caregivers [11, 12], and other fields [10, 13, 14]. It was less explored in patients with renal disease and only one study investigated 319 renal transplant patients and found BF was present from the pre-transplant stage to the post-transplant stage [15]. The BF in patients during the illness can be represented as follows, such as seeking meaning in life, living in the present, paying more attention to health, and perceiving support from others. Studies have proved that BF can help patients improve their physical and mental health, and leads to higher life satisfaction [16].

BF is reported to be affected by a variety of factors. In terms of demographic factors, such as age, education level, and economic income, no consensus has been reached yet [17]. However, negative emotions such as anxiety and depression were negatively correlated with BF [18, 19]. Moreover, previous studies have reported that BF is positively correlated with social support [20], positive coping [21] and general self-efficacy [11] in patients. Specifically, the perceived social support of individuals may not be equal to the social support they actually receive [22], but positive coping may lead patients to more easily perceive social support, so as to more actively deal with negative events and improve BF [12]. Besides, social support and positive coping are respectively positive influencing factors of BF, but as we know, there is still no study has explored the relationship between BF, social support, and positive coping in HD patients. Based on the above, the hypothesis of this study is that the level of BF in HD patients is related with social support, self-efficacy and coping style, and social support plays an indirect effect between positive coping and BF.

This study aims to investigate the current situation of BF in patients with chronic kidney disease who are receiving HD, analyze its influencing factors, and provide a preliminary reference for improving the mental health of those patients and developing targeted nursing intervention measures.

## Materials and methods

### Study Design

This is a cross-sectional study to explore the influencing factors of BF in HD patients.

### Participants

All the subjects were patients who had received HD treatment for a long time and were willing to participate in this study in the hemodialysis center of a 3A hospital in Shanghai from March to September 2019.

The inclusion criteria were as follows: [1] Patients who are diagnosed with chronic kidney disease undergoing hemodialysis; [2] Age ≥ 18 years; [3] Who can conduct a conversation consciously; [4] Without hearing or cognitive impairments. The exclusion criteria were as follows: [1] Patients who received kidney transplantation; [2] Patients who are in a critical condition; [3] Patients with a functional or organic mental illness; [4] Those who do not cooperate after explanation by the investigator.

A total of 17 independent variables were included in this study. The sample size was determined to be at least 204 based on the principle that 5–10 times the number of independent variables [23] and a 20% loss rate. A total of 260 questionnaires were handed out by convenience sampling, and 246 valid questionnaires were collected (effective rate = 94.6%). Among those invalid questionnaires, not all items or scales were completed.

### Measures

1. General Information Questionnaire.

Self-designed general information questionnaire included general demographic data on gender, age, religious belief, marital status, education level, medical insurance, and disease-related data on self-perceived disease severity, understanding of degree, and duration of HD.

(2) Benefit Finding Scale, BFS.

Benefit Finding Scale was developed by our research team [24] to measure the BF. The scale comprised 26 items with six dimensions, including *Spiritual Growth, Appreciation of Living and Life, Awareness of Social Support, Personal Growth, Altruistic Behavior*, and *Health behavior changes*. Each item is responded to on a 5-point Likert scale ranging from 0 to 4 (from “not at all” to “extremely”), with higher total scores indicating more BF experiences. This scale has been applied to 1007 patients with chronic diseases at least, which showed good utility [13, 14, 25]. The Cronbach’s α coefficient of the scale was 0.948 and the coefficients of each dimension were 0.700, 0.864, 0.776, 0.894, 0.859 and 0.838 in this study.

(3) Perceived Social Support Scale, PSSS.

It was developed by Blumenthal et al. in 1987 and used to measure the degree of social support an individual feels from various sources [26]. In this study, we applied its Chinese version [27], and it contains 12 items with three dimensions, including family support, friend support, and other support. The response options were on a 7-point Likert scale, ranging from “strongly disagree” (1 point) to “strongly agree” (7 points). A higher total score indicates a higher level of perceived support. The Cronbach’s α coefficient of the scale was 0.937 and the coefficients of each dimension were 0.914, 0.873 and 0.882 in this study.

(4) General Self-efficacy Scale, GSES.

The scale was originally developed by Schwarzer and Born [28], has been translated into several languages and is widely used in the world. We applied the Chinese version of the scale translated and revised by Wang et al. in 2001 in this study, which is a single-dimension scale with ten items [29]. Each item was on a 4-point Likert scale (1 = completely incorrect, 2 = relatively correct, 3 = mostly correct, and 4 = completely correct). The Cronbach’s α coefficient of the scale was 0.923 in this study.

(5) Simplified Coping Style Questionnaire, SCSQ.

It was compiled by Xie [30], with 20 items in 2 dimensions, including positive coping and negative coping. Each item is responded to on a 4-point Likert scale ranging from 0 to 3, with higher total scores indicating the participant tends to adopt this coping style. The scale is widely used in the Chinese clinical field. The Cronbach’s α coefficient of Simplified Coping Style Questionnaire in this study was 0.854, and of positive coping and negative coping dimension was 0.869 and 0.708 respectively.

### Data collection

This study complied with the Declaration of Helsinki for studies with human participants and was approved by the relevant Ethics Committee. Two researchers were trained in charge of questionnaires distribution uniformly before data collection, and selecting patients meeting the criteria after obtaining the consent of the hospital department leaders. The significance and purpose of the study were explained to the patients. Assurances of refusal or withdrawal from the study were provided to all participants without any negative consequences and signed written informed consent was obtained for this study.

### Data analysis

All analyses were performed using IBM SPSS software version 26 (IBM SPSS Inc. Chicago, Illinois, USA). The general demographic characteristics were described using counts and percentages. The scores of questionnaire scales were described using the median (interquartile range, IQR) rather than means and SD because data was not normally distributed. Differences between demographic variables were compared using analysis of variance (ANOVA) or non-parametric test (Mann-Whitney U test or Kruskal-Wallis H test) as appropriate. Spearman rank correlation coefficient was used to analyze the correlation among different questionnaire scale scores. Multiple linear regression analysis was performed with the BF score as the dependent variable and variables that were statistically significant (*p* < 0.05) in the univariate analysis of variance and correlation analysis as independent variables. The indirect effects of social support between positive coping and BF were analyzed by using hierarchical regression analysis and input method. The significance level was set to 0.05.

## Results

### Comparison of BF scores of patients with different demographic characteristics

The demographic characteristics information and results of univariate analysis about 246 HD patients investigated in this study are shown in Table 1. The age (Z = 6.151, *p* = 0.046), duration of HD (F = 4.608, *p* = 0.011), and understanding degree of HD (Z = 12.970, *p* = 0.005) differed at the BF level and were statistically significant.

**Table 1:** Demographic characteristics of participants (*N* = 246)

Categorical Variables	N (%)	M (IQR)	Z/F	p
Gender			2.018	0.155
Male	136 (55.3)	64 (20)		
Female	110 (44.7)	66 (20)		
Age			6.151	0.046
18–39	37 (15.0)	68 (23)		
40–59	95 (38.6)	64 (17)		
≥ 60	114 (46.3)	66 (18)		
Religious beliefs			0.001	0.972
Yes	17 (6.9)	67 (23)		
No	229 (93.1)	66 (19)		
Marital status			6.569	0.087
Married	202 (82.1)	66 (18)		
Unmarried	34 (13.8)	61 (13)		
Divorced	5 (2.0)	70 (39)		
Widowed	5 (2.0)	55 (40)		
Education			6.650	0.084
Primary or lower	11 (4.5)	56 (16)		
Junior high	75 (30.5)	66 (21)		
Senior high	78 (31.7)	63 (17)		
College or higher	82 (33.3)	67.5 (21)		
Form of medical security			5.032	0.169
Urban medical insurance	197 (80.1)	66 (19)		
Rural medical insurance	26 (10.6)	65 (28)		
Free medical treatment	11 (4.5)	57 (28)		
Other insurance	12 (4.9)	68 (31)		
Duration of hemodialysis (HD)			4.608	0.011
< 1 year	36 (14.6)	63 (27)		
1–5 years	97 (39.4)	67 (18)		
> 5year	113 (45.9)	65 (19)		
Self-assessment of disease severity			3.590	0.309
Mild	20 (8.1)	70 (18)		
Moderate	153 (62.2)	64 (18)		
Serious	62 (25.2)	66 (20)		
Very serious	11 (4.5)	66 (49)		
Understanding degree of HD			12.970	0.005
No understanding	6 (2.4)	57 (16)		
Partial understanding	156 (63.4)	64 (17)		
Adequate understanding	70 (28.5)	70 (25)		
Complete understanding	14 (5.7)	66 (20)		

M = median of BF score; IQR = interquartile range of BF score

### Scores of BF and each dimension

The median (interquartile range, IQR) BF score of 246 patients was 66 (IQR = 19), with a score range of 0 to 104, and the scores of each dimension and item were shown in Table 2.

**Table 2:** The median scores of benefit finding and its six dimensions

Variable	Items	Range	M (IQR) (Scale/Dimension)	M (IQR) (Item)
BF	26	0 ~ 104	66 (19)	2.54 (0.73)
Spiritual growth	2	0 ~ 8	4 (2)	2.00 (1.00)
Appreciation of living and life	5	0 ~ 20	13 (5)	2.60 (1.00)
Awareness of social support	4	0 ~ 16	10 (4)	2.50 (1.00)
Personal growth	7	0 ~ 28	17 (7)	2.43 (1.00)
Altruistic behavior	3	0 ~ 12	8 (3)	2.67 (1.00)
Health behavior changes	5	0 ~ 20	13 (4)	2.60 (0.85)

M = median; IQR = interquartile range

### Correlation analysis between BF and other variables

The results of Spearman correlation analysis showed that BF was positively correlated with disease knowledge, self-efficacy, social support and its three dimensions, and positive coping. The results were statistically significant, as detailed in Table 3.

**Table 3:** Correlation analysis among medical variables

NO.	Variables	1	2	3	4	5	6	7	8
1	BF	1	-	-	-	-	-	-	-
2	Self-efficacy	0.286**	1	-	-	-	-	-	-
3	Social support	0.618**	0.272**	1	-	-	-	-	-
4	Family support	0.581**	0.158*	0.867**	1	-	-	-	-
5	Friend support	0.492**	0.375**	0.871**	0.632**	1	-	-	-
6	Other support	0.574**	0.259**	0.896**	0.678**	0.723**	1	-	-
7	Positive coping	0.455**	0.460**	0.446**	0.376**	0.402**	0.394**	1	-
8	Negative coping	0.023	0.284**	-0.047	-0.172**	0.038	-0.028	0.340**	1

* *p*<0.05, ** *p*< 0.01

### Analysis of the influencing factors of BF

The assignment of the respective variables is shown in Table 4. The results showed that eight variables were included in the influence factor model (*p* < 0.05) and could collectively explain 43.8% of the total variation in BF, as shown in Table 5.

**Table 4:** Coding independent variables

Independent Variables	Methods of Coding
Age	1="18–39”;*2="40–59">3="≥60”
Duration of HD	*1=“<1 year”;2="1–5 years">3=”>5 years”
Knowledge of HD	*1="No understanding”;2="Partial understanding">3="Adequate understanding">4="Complete understanding”
Self-efficacy	Original numerical value
Family support	Original numerical value
Friend support	Original numerical value
Other support	Original numerical value
Positive coping	Original numerical value

* Reference group

**Table 5:** The multiple linear regression results of benefit finding

Independent Variables	Unstandardized Coefficients		Standardized Coefficients		t	p
	β	Std. Error				
Constant	4.455	4.814		-	0.925	0.356
Family support	1.116	0.220		0.323	5.071	< 0.001
Positive coping	0.441	0.152		0.173	2.904	0.004
Other support	0.746	0.240		0.207	3.103	0.002
Self-efficacy	0.360	0.145		0.135	2.475	0.014
Age 1	6.732	2.379		0.149	2.830	0.005
Age 3	3.748	1.766		0.116	2.122	0.035
Duration of HD 2	7.005	2.420		0.212	2.895	0.004
Duration of HD 3	5.018	2.355		0.155	2.131	0.034

F = 24.850, Adjusted R²=0.438, *p* < 0.001

Age 1="18–39”, Age3="≥60”; Duration of HD 2="1–5 years”, Duration of HD 3=”>5 years”

### Indirect effects of social support

As shown in Table 6, the regression analysis was first conducted with social support as the dependent variable, control variable as the first-tier predictor variable, and positive coping as the second-tier predictor variable. The results showed that β = 0.910 (*p* < 0.001) and R² = 0.482. Then, the regression analysis was conducted with BF as the dependent variable, control variable as the first-tier predictor variable, and positive coping as the second-tier predictor variable. The results showed that β = 1.130 (*p* < 0.001) and R² = 0.447. Finally, regression analysis with BF as the dependent variable, control variable as the first-tier predictor variable, and positive coping and social support as the second-tier predictor variable showed that β = 0.518 (*p* = 0.001) and R² = 0.624. The above results indicate that the indirect effect of social support in the BF of HD patients was well established, accounting for 54.1% of the total effect.

**Table 6:** The indirect effect of social support on positive coping and BF

Step	Dependent Variable	Independent Variable	β	β’	R	R²	t	p	F	p
1	Social support	(Control variable) Positive coping	- 0.910	- 0.483	0.482	0.232	- 8.441	- < 0.001	24.397	< 0.001
2	BF	(Control variable) Positive coping	- 1.130	- 0.444	0.447	0.200	7.596	< 0.001	20.158	< 0.001
3	BF	(Control variable) Positive coping Social support	- 0.518 0.672	- 0.204 0.497	0.624	0.390	- 3.500 8.659	- 0.001 < 0.001	38.482	< 0.001

Control variable: Age, Duration of HD

## Discussion

### BF in HD patients

The level of BF in HD patients needs further attention and help to improve. The results of this study showed that the median (interquartile range, IQR) BF score of HD patients was 66 (IQR = 19), a lower level especially compared with the older adults with chronic diseases (78.85 ± 16.70) [13] and stroke patients (97.47 ± 17.64) [14] in China. On the six dimensions of the scale, *Altruistic behavior* and *Appreciation of living and life* showed the higher scores, and *Spiritual growth* dimension had the lowest score. The possible reason for BF lower scores may be heterogeneity of population or disease, to be specific, the average age of participants in our study was younger than theirs, and some studies on cancer survivors had proved that elderly adults showed more BF [10, 18]. In addition, HD patients have to go to the hospital three or more times a week for dialysis, and it takes 4 to 6 h more once a time, which means HD patients have a more tremendous impact on daily life than other chronic diseases. patients showed positive psychological changes after HD, which were similar to previous studies of post-traumatic growth in HD patients, and this positive change is similar to the concept of *Personal Growth* and *Appreciation of Living and Life* [31]. Altruistic behavior can’t happen without the patient’s environment in China, a dialysis room is usually big and composed of more than six beds, which provides convenient environmental conditions for the occurrence of altruistic behavior. In such a multi-person dialysis room environment, HD patients can interact with others, because of the similar conditions, their psychological distance is narrowed, and they are more willing to communicate with each other [24]. In the process of helping others, people can often gain self-identity and value [32]. For example, Patients tend to share disease-related knowledge and encourage each other while receiving dialysis treatment. Other researchers have linked altruism to meaning in life, and people have a more intuitive sense of value, purpose, and direction when helping others [33]. For *Spiritual growth* dimension, what we want to evaluate is the patient’s mentality of recognizing the inevitability of the occurrence and accepting of the process HD. However, as the time of HD patients increases and their physical conditions deteriorate, most of them refuse to discuss or think about death, and cannot accept the fact that they are ill and eventually die after long-term hemodialysis [34]. At the same time, people are still sensitive to the topic of death in Chinese culture, and most of them take an avoidance attitude [35]. Therefore, in the investigation of this study, HD patients scored the lowest in spiritual dimension. What’s more, in this study, only 17 (6.9%) patients were religious, religious belief and spirituality in current studies are often assessed using an entry on religion (In research, religion and spirituality are often assessed with a single item on religious affiliation.) [36], This may partly explain the lowest spiritual dimension scores in HD patients in this study.

### Influencing factors of BF in HD patients

However, in this study, we found that the BF of HD patients was related to their Age, Duration of HD and Understanding degree of HD (*p* < 0.05). The results of this study showed that ages from 18 to 39 in HD patients presented the highest BF level, which was in line with Lv et al.(2017) for chemotherapy-phase breast cancer patients [37]. The second highest BF level was seen in the older group (≥ 60 years old), and the middle-aged group (40–59 years) had the lowest BF scores. According to the analysis of general demographic information, it can be assumed that the reasons may be that younger patients had fewer complications, healthier bodies, and intact family structures, which make them appreciate more of daily life in their lives, change unhealthy habits and feel more confident about their future. In addition, they were willing to accept new things. Middle-aged people faced a heavier burden in life than older adults [38]. *Duration of HD* was another significant factor affecting BF level found in this study, unlike the result of De Vries et al. (2019) in renal transplant patients [15], which no between-person effect was found for time on dialysis on BF. The consideration may be such patients have reached the worst critical stage of disease, when they are more concerned about how to prolong their lives but less about getting psychological benefits from the disease. The results showed that the BF score of HD patients with dialysis duration < 1 year was the lowest score of 63 (IQR = 27), they would be full of fear and anxiety when just accept dialysis treatment at the beginning, resulting in fewer benefits from the disease [39]. The HD patients with 1–5 years dialysis time had the highest BF scores of 67 (IQR = 18). With the prolongation of dialysis time, the patients further get the knowledge and skills related to HD, gradually get used to dialysis every time. In addition, patients also know more friends who have the same experience (on dialysis), and encourage and help each other. At this stage, their psychology develops in a positive direction, and BF level is improved. Patients with dialysis duration > 5 years BF score was 65 (IQR = 19), after 5 years of dialysis, complications increased [40] and negative emotions repeatedly appeared [5, 41], then BF scores decreased.

*Self-Efficacy* was a promoting factor of BF in our study, which was consistent in Chinese patients with cancer and their family caregivers [42]. It is a strong perception and a source of motivation that drives individuals to overcome challenges and ultimately succeed [43]. Previous studies have shown that there is a strong correlation between self-efficacy and coping strategies [44], and are more inclined to adopt positive coping styles when face stressful events [45]. *Social support* in HD patients was another positive factor, which had been replicated in previous studies [12, 18], all the dimensions scores of social support and BF were positively correlated respectively, with family support and other support eventually entering the influence factors model of BF. The reason might be that family support was one of the strongest emotional and material support for HD patients, which was related to Chinese traditional family values. Friends were the main component of patients’ social relations, and medical staff was their medical trust subjects. A qualitative study found that positive interactions with family members and friends could make HD patients experience a greater sense of meaning in life and hope for the future [46], the feelings mentioned above are all similar to the concept of BF. Community and public groups provided them with the support of social relations, such as patient communication organizations, relevant protection policies, and companies that support patients to continue working. Those help patients to enhance confidence, bring hope in life, face the disease more positively, then improve their BF level. The results showed that there was a positive correlation between *Positive Coping* and BF, which was similar to the results in female breast cancer survivors [9]. Social Support was a mediating variable between Positive Coping and BF and played an indirect effect. We considered that HD patients receiving insufficient internal support because of tremendous stress, and they tended to seek external help. Patients who coped with HD positively might receive more social support and feel internally not struggling with the disease alone. Then they could rationalize their mindset and had more confidence in mobilizing the patients to adopt a positive approach to the disease, thus demonstrating a higher level of BF.

### Strengths and limitations

This study was the first to explore the cross-sectional study of HD patients with BF experience and investigated the demographic factors, duration of HD, understanding degree of HD, self-efficacy, social support, and disease coping as possible predictors and the relationship of the BF, also explored the Social Support as an indirect effect between the Positive Coping with BF. It provided a reference for future more scientific and reasonable psychological promotion programs for HD patients.

The results of the study and conclusion should be carefully referenced. First, the research used convenient sampling in a 3 A hospital in Shanghai, which may not be a good representative of the BF status of HD patients in other cities in China, and a larger sample and multi-center investigation were still needed. In addition, BF was a psychological change indicator with the further dynamic development of the disease course, which required strict longitudinal study to determine the trajectory.

## Conclusion

Our study reported that although the BF of HD patients investigated was not satisfactory, HD patients could experience positive changes from disease. Medical staff can attach importance to the influence factors of BF by providing patients with adequate emotional assistance and information to help the patients enhance self-efficacy, perceive social support and learn positive coping in dealing with problems during dialysis and then improving quality of life. In the future, the mechanism of patients’ BF and the main influencing factors can be further focused on and explored according to the influencing factors of BF, and formulated intervention strategies to provide a scientific basis for promoting their BF.

### Electronic supplementary material

Below is the link to the electronic supplementary material.

Supplementary Material 1

## Data Availability

All data generated or analysed during this study are included in this published article and its supplementary information files.
